# Supplementary material for: Multi‐dataset identification of innovative feature genes and molecular mechanisms in keratoconus
Source: J Cell Mol Med. 2024 Sep 19;28(18):e70079. doi: 10.1111/jcmm.70079 (PMC11412914; doi:10.1111/jcmm.70079)
Supplement: Supplementary file 2 — Material S2. [file JCMM-28-e70079-s002.zip › 7.GSEA analysis/5.RNF217-AS1/KEGG_DNA_REPLICATION.html]

Details for gene set KEGG\_DNA\_REPLICATION[GSEA]

|  || Dataset | RNF217-AS1.group.cls#h\_versus\_l |
| Phenotype | group.cls#h\_versus\_l |
| Upregulated in class | l |
| GeneSet | KEGG\_DNA\_REPLICATION |
| Enrichment Score (ES) | -0.45193666 |
| Normalized Enrichment Score (NES) | -1.5257813 |
| Nominal p-value | 0.038306452 |
| FDR q-value | 0.3563103 |
| FWER p-Value | 0.725 |
Table: GSEA Results Summary

  

Fig 1: Enrichment plot: KEGG\_DNA\_REPLICATION      
 Profile of the Running ES Score & Positions of GeneSet Members on the Rank Ordered List

  

| SYMBOL | TITLE | RANK IN GENE LIST | RANK METRIC SCORE | RUNNING ES | CORE ENRICHMENT || 1 | RPA4 | na | 732 | 0.481 | 0.1032 | No |
| 2 | POLE | na | 10398 | 0.080 | -0.1549 | No |
| 3 | DNA2 | na | 11951 | 0.056 | -0.1851 | No |
| 4 | POLD4 | na | 14819 | 0.021 | -0.2623 | No |
| 5 | PRIM2 | na | 16280 | 0.006 | -0.3029 | No |
| 6 | POLD1 | na | 16368 | 0.005 | -0.3041 | No |
| 7 | SSBP1 | na | 17026 | -0.002 | -0.3225 | No |
| 8 | RNASEH2B | na | 17994 | -0.011 | -0.3476 | No |
| 9 | POLE4 | na | 18097 | -0.012 | -0.3475 | No |
| 10 | MCM7 | na | 21720 | -0.048 | -0.4397 | Yes |
| 11 | POLD3 | na | 21754 | -0.048 | -0.4282 | Yes |
| 12 | RNASEH1 | na | 22324 | -0.054 | -0.4307 | Yes |
| 13 | POLE3 | na | 22462 | -0.056 | -0.4203 | Yes |
| 14 | POLD2 | na | 23076 | -0.063 | -0.4217 | Yes |
| 15 | RNASEH2C | na | 23280 | -0.065 | -0.4108 | Yes |
| 16 | RPA1 | na | 23365 | -0.066 | -0.3960 | Yes |
| 17 | POLA1 | na | 23390 | -0.067 | -0.3795 | Yes |
| 18 | RFC5 | na | 23446 | -0.067 | -0.3637 | Yes |
| 19 | RFC1 | na | 23605 | -0.070 | -0.3502 | Yes |
| 20 | RPA2 | na | 23725 | -0.071 | -0.3353 | Yes |
| 21 | RNASEH2A | na | 24015 | -0.076 | -0.3241 | Yes |
| 22 | PCNA | na | 24885 | -0.089 | -0.3263 | Yes |
| 23 | RPA3 | na | 24916 | -0.089 | -0.3042 | Yes |
| 24 | LIG1 | na | 24995 | -0.090 | -0.2832 | Yes |
| 25 | RFC2 | na | 25014 | -0.091 | -0.2603 | Yes |
| 26 | RFC4 | na | 25395 | -0.097 | -0.2463 | Yes |
| 27 | MCM3 | na | 25627 | -0.100 | -0.2270 | Yes |
| 28 | MCM5 | na | 27348 | -0.133 | -0.2423 | Yes |
| 29 | RFC3 | na | 27480 | -0.136 | -0.2109 | Yes |
| 30 | MCM4 | na | 28135 | -0.152 | -0.1905 | Yes |
| 31 | MCM6 | na | 29532 | -0.192 | -0.1812 | Yes |
| 32 | PRIM1 | na | 29707 | -0.197 | -0.1354 | Yes |
| 33 | MCM2 | na | 30570 | -0.226 | -0.1019 | Yes |
| 34 | POLA2 | na | 30950 | -0.240 | -0.0510 | Yes |
| 35 | FEN1 | na | 31474 | -0.262 | 0.0017 | Yes |
| 36 | POLE2 | na | 32955 | -0.357 | 0.0511 | Yes |
Table: GSEA details [plain text format]

  

Fig 2: KEGG\_DNA\_REPLICATION      
 Blue-Pink O' Gram in the Space of the Analyzed GeneSet

  

Fig 3: KEGG\_DNA\_REPLICATION: Random ES distribution      
 Gene set null distribution of ES for **KEGG\_DNA\_REPLICATION**
